# A Review of the CFD Modeling of Hydrogen Production in Catalytic Steam Reforming Reactors

**DOI:** 10.3390/ijms232416064

**Published:** 2022-12-16

**Authors:** Nayef Ghasem

**Affiliations:** Department of Chemical & Petroleum Engineering, United Arab Emirates University, Al Ain P.O. Box 15551, United Arab Emirates; nayef@uaeu.ac.ae

**Keywords:** catalysis, CFD, reforming, steam reforming, hydrogen, catalytic, conversion of CO_2_

## Abstract

Global demand for alternative renewable energy sources is increasing due to the consumption of fossil fuels and the increase in greenhouse gas emissions. Hydrogen (H_2_) from biomass gasification is a green energy segment among the alternative options, as it is environmentally friendly, renewable, and sustainable. Accordingly, researchers focus on conducting experiments and modeling the reforming reactions in conventional and membrane reactors. The construction of computational fluid dynamics (CFD) models is an essential tool used by researchers to study the performance of reforming and membrane reactors for hydrogen production and the effect of operating parameters on the methane stream, improving processes for reforming untreated biogas in a catalyst-fixed bed and membrane reactors. This review article aims to provide a good CFD model overview of recent progress in catalyzing hydrogen production through various reactors, sustainable steam reforming systems, and carbon dioxide utilization. This article discusses some of the issues, challenges, and conceivable arrangements to aid the efficient generation of hydrogen from steam reforming catalytic reactions and membrane reactors of bioproducts and fossil fuels.

## 1. Introduction

A modern approach is currently being considered to reduce greenhouse gas emissions for power generation and fuel production for the automotive sector as well as for domestic applications. As a result, the research on the feasibility of applying renewable energy sources in the current energy scenario is gradually increasing [1]. This is by following the renewable energy approach, which mainly involves using raw materials derived from biomass and combining energy generated through clean sources and traditional energy generation systems [2]. Recently, interest has been focused on the pathway from bioethanol reformation to catalyzing hydrogen production through sustainable reforming and carbon dioxide utilization. It leads to the use of the produced carbon dioxide for various purposes, passing through the stage of transformation between water and gas. The latest approaches presented in the literature are reviewed, showing that they can successfully produce green and sustainable hydrogen. This can represent an energy storage technology; this hydrogen can be used to convert carbon dioxide into hydrocarbons, giving added value to carbon dioxide [3,4,5,6].

**Figure 1:**
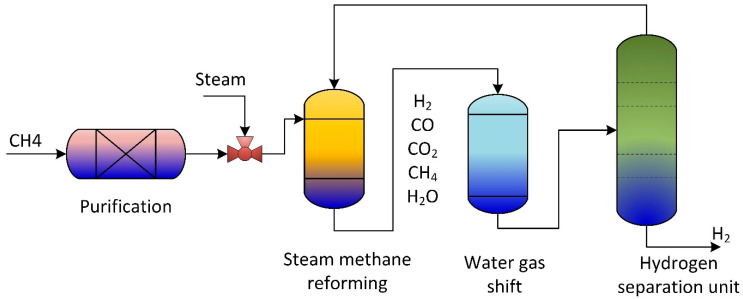
Schematic of traditional methane reforming unit for hydrogen production.

According to the International Energy Agency (IEA), in 2002, two-thirds of all fossil fuel combustion and CO_2_ emissions were produced from the transportation sector [7]. Due to current environmental care and government regulations, a new clean energy resource is crucial. Hydrogen gained popularity as an environmentally friendly energy and fuel source. Therefore, hydrogen fuel cells in transportation are supported as they effectively have zero emissions. Small-scale methane steam reformers are commercially available for small production rates; they still require high throughput, high reliability, and high purity. The steam reforming process is used to produce hydrogen gas from hydrocarbon gas via catalytic reactions. Figure 1 is a general steam reforming process on the industrial scale. The reformer (steam methane reformer) is the primary unit in the process and consists of the tube and furnace side. Combustion of typical air, hydrogen, methane, and carbon oxides took place on the furnace side. The generated heat is utilized to heat the reforming tubes by exchanging radiative heat. Catalytic reactions occur in the reforming tubes, where methane and steam are converted into hydrogen, CO, and CO_2_.

Therefore, previous studies to measure the dependency of materials performance in the steam reforming process used experimental [8] or computational fluid dynamics (CFD) modeling and simulation approach [9]. Carbon deposition during thermal catalytic cracking resulted in catalyst deactivation, a crucial phenomenon that causes a drop in the reaction rate and hence reactor blockage circumstance. The CFD model explored the coke deposition on the catalyst’s surface and considered the variation of catalyst properties with time during coke formation on the catalyst particle and in the packed bed reactor. The developed CFD modeling studies the interaction of catalyst activity on reaction performance during coke formation [10]. The most used production method is catalytic steam methane reforming in a fixed bed [11]. Various membrane and fixed bed reactors are used to produce hydrogen from biomass and fossil fuels. Improving steam methane reforming reactor performance is crucial to increase throughput and reduce energy losses. Therefore, efficient computational fluid dynamics (CFD), analysis, and developments are essential. Table 1 lists the most common reactors and software packages used in the CFD simulation of the steam methane reforming reactors. The present work reviews the CFD models developed for steam reforming reactors utilized in hydrogen production and the software packages used in the CFD simulation of the steam reforming unit to increase system productivity.

## 2. Mathematical Models

Various mathematical models were developed for hydrogen production in steam reformers with and without membranes. The following sections reviewed the most widely used CFD models for different configuration processes employed for hydrogen production in traditional and membrane reactors.

### CFD Modeling Equations

The CFD models generally consider the following assumptions: steady-state, ideal gas behavior, laminar flow, constant physical properties, and no-slip conditions. Sample mathematical equations are shown below [37]:

Continuity equation:

(1) $$\frac{\partial \left(\rho u\right)}{\partial x}+\frac{\partial \left(\rho v\right)}{\partial y}=0$$

Momentum equations:

(2) $$\frac{\partial \left(\rho uu\right)}{\partial x}+\frac{\partial \left(\rho uv\right)}{\partial y}+\frac{\partial p}{\partial x}-\frac{\partial }{\partial x}\left[2\mu \frac{\partial u}{\partial x}-\frac{2}{3}\mu \left(\frac{\partial u}{\partial x}+\frac{\partial v}{\partial y}\right)\right]-\frac{\partial }{\partial y}\left[\mu \left(\frac{\partial u}{\partial y}+\frac{\partial v}{\partial x}\right)\right]=0$$

(3) $$\frac{\partial \left(\rho uv\right)}{\partial x}+\frac{\partial \left(\rho vv\right)}{\partial y}+\frac{\partial p}{\partial x}-\frac{\partial }{\partial y}\left[\mu \left(\frac{\partial v}{\partial x}+\frac{\partial u}{\partial y}\right)\right]-\frac{\partial }{\partial x}\left[2\mu \frac{\partial v}{\partial y}-\frac{2}{3}\mu \left(\frac{\partial u}{\partial x}+\frac{\partial v}{\partial y}\right)\right]=0$$

Energy equation:

(4) $$\frac{\partial \left(\rho uh\right)}{\partial x}+\frac{\partial \left(\rho vh\right)}{\partial y}+\frac{\partial }{\partial x}\left(\rho \sum_{k=1}^{K_{g}}Y_{k}h_{k}V_{k,x}-\lambda_{g}\frac{\partial T}{\partial x}\right)+\frac{\partial }{\partial y}\left(\rho \sum_{k=1}^{k_{g}}Y_{k}h_{k}V_{k,y}-\lambda_{g}\frac{\partial T}{\partial y}\right)=0$$

Gas-phase species equation:

(5) $$\frac{\partial \left(\rho uY_{k}\right)}{\partial x}+\frac{\partial \left(\rho vY_{k}\right)}{\partial y}+\frac{\partial }{\partial x}\left(\rho Y_{k}V_{k,x}\right)+\frac{\partial }{\partial y}\left(\rho Y_{k}V_{k,y}\right)-\dot{\omega_{k}}W_{k}=0,\;where\;k=1,\ldots ,k_{g}$$

The heat of the reaction:

(6) $$CH_{4}+H_{2}O\;↔CO+3H_{2},\;\;\;∆H_{r}=206.6\frac{kJ}{mol}$$

(7) $$CO+H_{2}O\;↔CO_{2}+3H_{2},\;\;\;∆H_{r}=-41.1\frac{kJ}{mol}$$

where the heat of reaction ($∆H_{r}$) is at a temperature of 25 °C; in this kinetic model, the coke decomposition is negligible since the steam concentration in the feed stream is marginally more than the stoichiometric ratio.

(8) $$r_{MSR}=k_{MSR}\left(p_{CH_{4}}p_{H_{2}O}-\frac{p_{CO}p_{H_{2}}^{3}}{k_{eqMSR}}\right)$$

(9) $$r_{WGS}=k_{WGS}\left(p_{CO}p_{H_{2}O}-\frac{p_{CO_{2}}p_{H_{2}}}{k_{eqWGS}}\right)$$

Solving for the rate of reaction using the Arrhenius equation:

(10) $$k_{MSR}=2395\exp\left(\frac{-231266}{R_{u}T}\right)$$

(11) $$k_{WGS}=0.0171\;exp\left(\frac{-103191}{R_{u}T}\right)$$

(12) $$k_{eqMSR}=1.027\times 10^{10}\exp\left(-0.25Z^{4}+0.3667Z^{3}+0.58Z^{2}-27.134Z+3.28\right)$$

(13) $$k_{eqMSR}=\exp\left(-0.2935Z^{3}+0.6351Z^{2}+1.1788Z+3169\right)$$

(14) $$Z=\frac{1000}{T}-1$$

where r stands for rate of reaction, thus with subscript membrane shift reaction (*MSR*) and water–gas shift reaction (*WGS*) means the chemical reaction rate, the membrane shift reaction (*MSR*) $\left(\frac{mol}{m^{3}s}\right)$, and rate of reaction *WGS* $\left(\frac{mol}{m^{3}s}\right)$, respectively. Subsequently, K is the constant of reaction, $K_{eq}$ is the equilibrium constant of the reaction, $p_{i}$ is species i, partial pressure (Pa), the temperature (*T*) in Kelvin, and $R_{u}$ is the universal gas constant, which equals $8.314\frac{J}{mol\;K}$.

## 3. Types of Membrane Reactors

A membrane reactor is a piece of chemical equipment that combines a catalyst-filled reaction chamber with a membrane for adding reactants or removing reaction products. When the reaction involves some form of catalyst, membrane reactors are more commonly used [38,39,40]. Chemical reactors that use membranes are often referred to as membrane reactors [41]. There are two main types of membrane reactors: the catalytic membrane reactor and the inert membrane reactor. A catalytic membrane reactor has a membrane made of a catalyst-containing material, which means that the membrane itself takes part in the reaction; some of the reaction products pass through the membrane and exit the reactor on the permeable side [42,43]. By contrast, the inert membrane reactor allows catalyst pellets to flow with the reactants on the feed side [44,45]. It simply acts as a barrier for the reactants and some products and the membrane does not participate in the reactions directly [46].

### 3.1. Reformer with a Variable Number of Membranes

**Figure 2:**
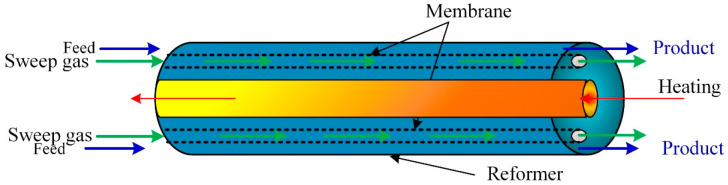
Schematic of a membrane reactor with multiple membrane tubes [47].

**Figure 3:**
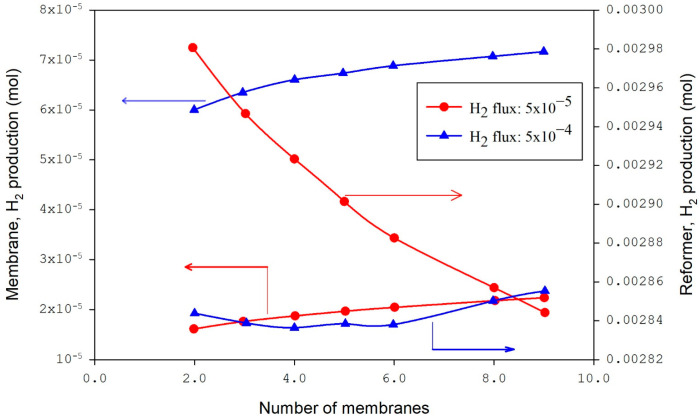
Hydrogen yield in membrane and reformer bed with variable fluxes of hydrogen [47].

**Figure 4:**
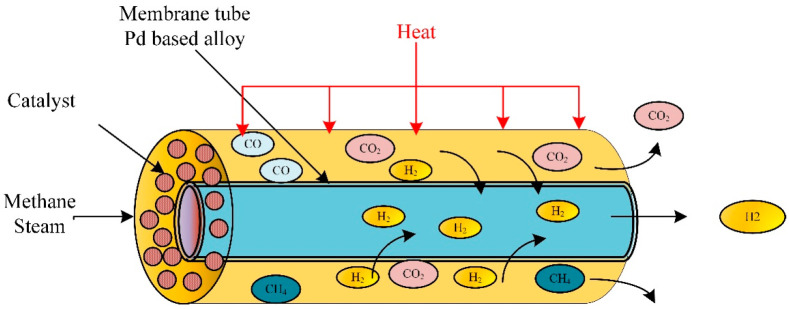
Membrane-based methane steam reforming for hydrogen production.

**Figure 5:**
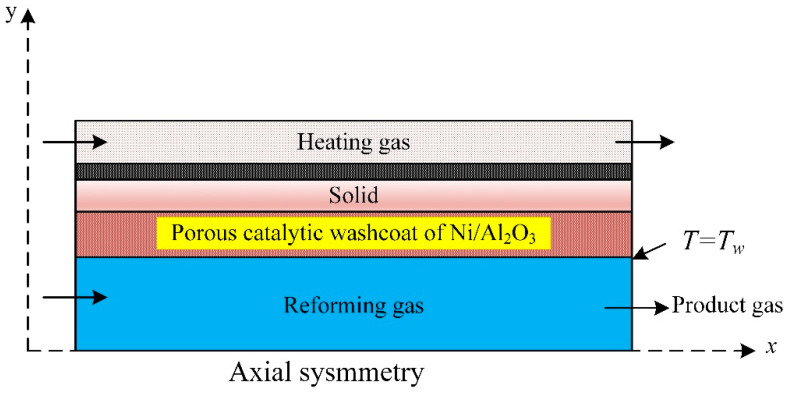
Microchannel reactor for methane steam reforming simulated with CFD [65].

**Figure 6:**
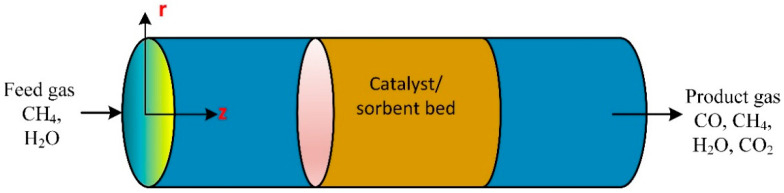
A schematic diagram of fixed bed catalytic reactor [72].

**Figure 7:**
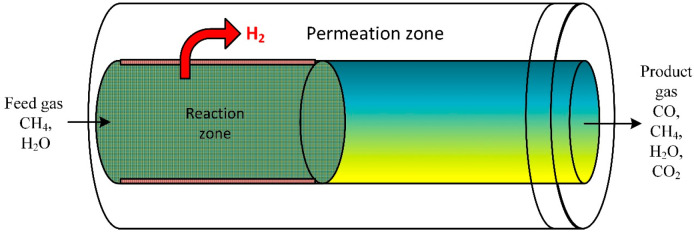
Schematic of a packed bed reactor assisted by membrane [85].

**Figure 8:**
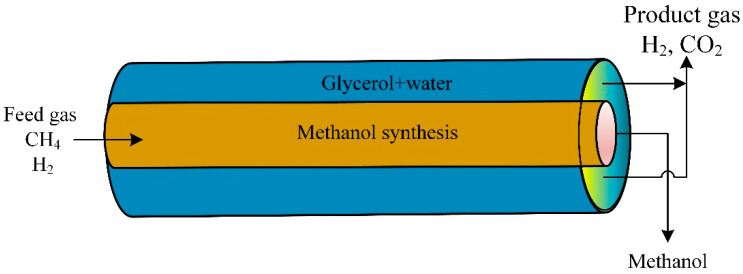
Schematic diagram of thermally double-coupled reactor [90].

**Figure 9:**
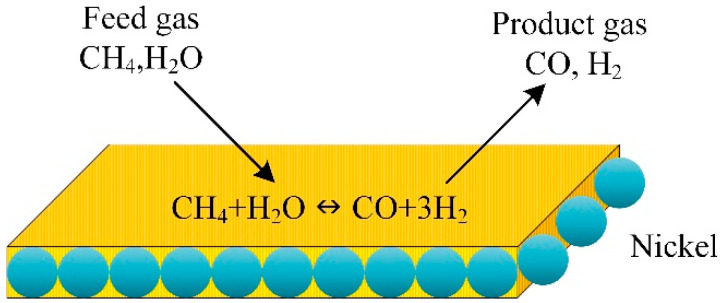
Schematic illustration of methane catalytic steam reforming [91].

The CFD modeling and simulation approach examines the performance of methane reforming of CO_2_ in a membrane reactor and the impact of the number of membranes (Figure 2, Figure 3, Figure 4, Figure 5, Figure 6, Figure 7, Figure 8 and Figure 9) at a fixed distance between the membrane and reactor centers on hydrogen production efficiency. Results revealed that hydrogen permeation increased with the number of membranes (Figure 2); by contrast, the conversions of carbon dioxide and methane and the production of methane decreased in the reformer bed [47]. Figure 3 shows that at a flux of 5 × 10^−4^, the hydrogen in the membrane slightly increased with the increased number of membranes and had an insignificant effect in the reformer. At lower flux values (5 × 10^−5^), the reformer hydrogen dropped, and the membrane hydrogen was unchanged with the number of membranes.

A similar modeling approach has been developed to describe the performance of a coated wall microreactor for methanol vapor reformation and hydrogen production. The forecast of the CFD model shows good agreement with the observed data. The 3D CFD model executed with COMSOL Multiphysics concluded that methane conversion is proportional to the increase in the inlet temperature of the heating tubes, and the ratio of CH_4_/H_2_O is best at 0.2, whilst porosity below 0.35 does not affect the conversion of methane [48]. Cylinder height = 150 mm, wall thickness = 3 mm, Brinkman equation, incompressible, mass: Maxwell–Stefan, energy steady state. A catalytic methane steam reforming of bio-oil pyrolysis in a fixed bed catalytic reactor utilized a low-cost material such as dolomite to produce hydrogen. The modeling generated predictions revealed that the nickel catalyst is efficient in reaching 100% conversion of methane. In another study, the carbon deposition in the reactor revealed that the bio-oil steam reforming in a multi-stage packed column reforming system is essential [49]. Bio-oil and its derivatives are employed to produce hydrogen by catalytic steam reforming. Mixtures of bio-oil contain a considerable number of compounds generated by the fast pyrolysis of biomass, revealing that an increase in the steam-to-carbon molar ratio at high temperatures increases the yield and the carbon conversion of H_2_ and CO_2_ [50].

### 3.2. Catalyst Decomposition

Hydrogen is produced from a catalytic steam reforming of biodiesel byproducts such as glycerol. The performance modeling analysis of hydrogen production from glycerol (biodiesel byproduct) catalytic steam reforming revealed that the high content of oxygen, impurities, and complex intermediates makes glycerol steam reforming a challenging process [51]. The model takes into account the thermal decomposition of the catalyst [52]. Hydrogen produced from steam reforming of aqueous fractions of bio-oil under catalysts prepared with different nickel contents revealed that an increase in the reaction temperature up to 750 °C improved the overall conversion and the hydrogen yield. By contrast, a further increase in the temperature above 750 °C loses the performance of the catalysts and causes the formation of carbon deposits. Hence, catalyst deactivation derivatives of bio-oil are an important biomass source to produce hydrogen obtained by steam reforming pyrolysis. Rhodium (Rh) as a noble metal proved to achieve high efficiency in improving the reaction compared with Ni-based catalysts with carbon deposition difficulties due to chemical poisoning. The reaction mechanisms and future scenarios in the catalyst development used in catalytic steam reforming [53]. Mathematical modeling developed of steam methane reforming conducted for industrial steam methane reforming taking into account catalyst inactivation. The study concluded that working under ideal conditions increases hydrogen production by about 11.6%. Additionally, the product’s process emission performance (ratio of hydrogen-to-carbon-dioxide) is 6.72 for customary conditions and 7.03 for enhanced conditions [54].

Using the density functional theory (DFT), activation energy for methane decay gradually decreases as the reaction continues to the benzene-absorbed state. It increases significantly in the step producing carbon and hydrogen, preventing the further deterioration of benzene into carbon and hydrogen [55]. In contrast, the alternative of reactions with lower activation energy leading to CO gas progression facilitates the methane breakdown continuously and improves H_2_ gas growth [55]. A two-dimensional computational fluid dynamics (CFD) model shows that stable and efficient reactor operation is feasible at a fraction of second contact times with high conversion [56]. A lower steam-to-carbon ratio increases the power output at lower temperatures. In terms of conductivity, moderate-conductivity materials balance conversions and temperature, and lower-conductivity materials allow higher conversions and power outputs with high-temperature spots [56]. Fuel cell reformers improved by optimizing the reactors and heat exchangers [57]. The model development used the COMSOL software package to simulate the coupling of vapor and post-oxidation performance as an effective tool for complex chemical engineering phenomena.

The possibility of steam methane reforming to yield hydrogen through a thermally integrated microchemical process has been studied [37]. The study predicts that for a feasible design process, an improved catalyst performance is significant to the approach. Furthermore, meticulous design and application of the method are also essential to allow high thermal integration. Moreover, channel height plays a vital role in defining the effectiveness of heat exchange. A suitable balance of the flow rates of the reforming streams and combustibles is an important design principle. Lastly, the catalyst loading must be studied carefully to avoid insufficient reactant conversion or high-temperature spots. The rapid thermal decomposition of the corn stalk bio-oil produces hydrogen by the catalytic steam reforming supported by nickel/alumina [37].

### 3.3. Membrane-Based Reactor

A computational fluid dynamics (CFD) study of steam ethanol reforming above a nickel-based catalyst with crossflow configuration is accompanied to measure the influence of six parameters on ethanol conversion and H_2_ yield. The outcomes indicate that the higher the reaction pressure, the better the ethanol steam reforming (ESR) performance. Moreover, increasing the ratio of the tube diameter (D) to the width of the channel (W) or the thickness of the catalyst (T) to the tube diameter (D) ratio improves the ethanol conversion and hydrogen yield. The ethanol conversion has no significant change when the S/E ratio is above 4. Increasing the number of tubes boosts ethanol conversion; furthermore, the influence of the altered Reynolds number on the performance is insignificant. When the T/D ratio is 0.33, the ethanol conversion achieves around 100% [58]. CFD modeling of methylcyclohexane dehydrogenation in the membrane reactor revealed that increasing the reaction temperature leads to a significant increase in methylcyclohexane conversion in the membrane reactor. At the same time, it did not significantly affect the hydrogen recovery, which remained stable. Furthermore, an increase in pressure caused a slight decrease in the conversion. It increased the hydrogen recovery, and an increase in the sweep factor led to an improved dehydrogenation conversion process and hydrogen recovery. Moreover, an increase in feed molar flow rate induced decreased methylcyclohexane conversion and hydrogen recovery [59].

A comprehensive computational model developed and solved using COMSOL Multiphysics to predict the membrane performance was simulated using CFD. The membrane dehydrogenation reactor for hydrogen production from methylcyclohexane (Figure 4). After validating the model with experimental data, the model predicts the influence of the operating parameters on the membrane reactor performance. The results showed that the membrane reactor performance was better than the traditional equivalent reactor, achieved complete conversion of methylcyclohexane, and hydrogen recovery was around 96%.

### 3.4. Membrane-Based Fixed Bed Reactor

The rapid thermal decomposition of the corn stalk bio-oil produces hydrogen by steam catalytic reforming supported by nickel/alumina. The cerium upgraded the supported catalysts using a fixed bed reactor connected to a Fourier transform apparatus and a thermal conductivity meter. The results revealed a decrease in hydrogen production with an increase in space velocity, with a slight increase in the percentage of coke deposition [60]. An untreated water–gas shift reaction of biogas reformate took place in both membranes and fixed bed reactors to produce hydrogen. The CFD model was developed and used to study the effect of operating parameters on system performance. The CFD model predictions were validated with membrane and packed bed reactor experiments.

The attained conversion of the equilibrium reaction is at 2.95 gcat.h/(mol.time), and a 3.4 steam-to-carbon ratio; the CO counterflow configuration enhanced the CO conversion [61]. At 1 bar pressure and a temperature of 300 to 700 °C, the kinetic rate data are presented for the methane steam reforming linked with water–gas shift reactor over 18 wt% catalysts (NiO/Al_2_O_3_). A mathematical model was developed to describe the catalytic reaction in a packed bed reactor. Experimental results were used to validate the developed mathematical model. The model predictions were well in line with the experimental results. The authors used the model to study different operating factors on conversion ratio and hydrogen production [62]. Experiments were performed in a packed bed reactor with a single pellet string to investigate the catalytic methane steam reforming. The reaction was tremendously hot, and thermocouples were used to measure the temperature of the outlet gas stream, the inlet, and the outer and inner surfaces of the catalyst particle. The CFD simulations were compared to the experimental data and found to be in good agreement. The methane steam reforming reaction strongly influenced the temperature profile inside the reactor; initially, the temperature dropped slightly [63].

The acetic acid compound present in the latter and separated into lignin and aqueous is considered a perfect compound of liquid pyrolysis (bio-oil) to produce hydrogen by catalytic steam reforming. The process is economical and environmentally friendly. Various Ni catalysts were prepared with multiple nickel content. The effect of reduction time for catalyst particles, temperature, and the weight ratio of catalyst-to-acid were investigated [64].

### 3.5. Microchannel Plates Steam Reformer

A CFD model coupled with the kinetics of the methane steam reforming over Ni catalyst is used for the numerical analysis of methane steam reforming; a reactor consists of microchannel plates (Figure 5). The simulation predictions revealed that the reaction and the internal mass transfer governed the process performance and depended on the wash coat’s dimensions and structure. The heat coupling improved with increased wash coat pore size. Activity, price, and specified process demand control the wash coat properties [65].

Ethanol steam reforming over Rh–Pd/CeO_2_ catalytic in-wall reactors was conducted. A mathematical model was developed to describe the steam reforming process. The developed three-dimensional CFD non-isothermal model successfully simulated the hydrogen production under realistic conditions. The method attained 80% of maximum hydrogen yield at 1150 K temperature, 4 bar pressure, and a steam-to-carbon ratio of 3 [27]. A 2D mathematical model developed to describe the principles of steam reforming considered the interaction between catalyst, pressure drop, and heat transfer in the reactor’s design. Computation fluid dynamics modeling was conducted to understand the steam reforming reactors flow patterns, considering pressure drop, heat, and mass transfer. The developed model forms the basis of steam reformer modeling [66]. CFD modeling was developed to analyze the methanol steam reforming process, whilst reactions kinetic parameters were obtained experimentally. Methanol is an essential feed for hydrogen production for easy transportation and high energy density.

Several variables, such as the rate of hydrogen production, methanol conversion, and the concentration of the resulting mixture, were evaluated. CFD models considered variable reactor length, variable catalyst layer temperatures, space velocity, and vapor-to-carbon ratio [67]. Dimethyl ether (DME) steam reforming presented with the computational fluid dynamic (CFD) model was used to describe the DEME steam reforming in a circulating fluid (CF) bed reactor. The ANSYS software package solves the CFD model. The hydrogen yield is 59%, and DME conversions up to 87%. The product gas contains 72% hydrogen [68]. CFD modeling study of Pd membrane for methanol steam reforming reactors revealed that the Pd-based membranes are frequently used in the various applications with a high hydrogen production rate.

The drawback of the Pd membrane is the cost and low permeability of hydrogen, and these factors hindered the extensive scale development. The experimental data from the literature validated the CFD model predictions. The model is employed to investigate the temperature and reaction pressure on the performance of the membrane reactor in terms of methanol conversion, hydrogen yield, and the selectivity of carbon monoxide. The optimum simulation results are at 513 K and 10 bars [69]. The 3D mathematical model was developed to predict the chemical reaction and heat transfer inside the tubular reactor for the methane steam reforming unit. Methane conversion decreases with increased inlet flow rate (more reactants); by contrast, the efficiency of the energy storage increases and then drops once the methane conversion decreases [70]. The CFD model developed to describe methane steam reformer composed of mass, energy balances, and momentum studied the effects of inlet temperature of heating tubes, CH_4_/H_2_O inlet ratio, and heating tube configuration on the reactor performance. The predictions revealed that methane conversion increased with inlet temperature and the number of heating tubes in the reformer. The optimum methane-to-steam ratio was about 0.2. The CFD simulation predictions were in good agreement with experimental data from the literature. The simulation outcomes apply to the industrial scale of natural gas reformer [71].

### 3.6. Packed Bed Tubular Reactor

The unsteady-state 2D numerical was model developed and experimentally verified to study the influence of temperature on the sorbent of methane steam reforming in a packed bed tubular reactor with a fixed wall temperature of 600 °C (Figure 6). The reactor used a CaO sorbent and Ni/Al_2_O_3_ catalyst. The methane conversion and hydrogen molar fraction were 52.6% and 78.7%, respectively. The reaction of methane steam reforming was enhanced by the continuous removal of carbon dioxide through the reaction with CaO to produce CaCO_3_. Results revealed the conversion of CH_4_ and the H_2_ molar fraction increased with diameter [72]. Bio-oil pyrolysis in a catalytic steam reformer was used for hydrogen production in a fluidized bed reactor. The optimum hydrogen production took place at 700 °C. The steam-to-carbon molar ratio was 17. The catalyst decayed in a fluidized bed due to the NiO-sintered grain on the surface of the supporter, is the leading reason for the deactivation of the fresh catalyst [73]. Bio-oil aqueous fractions produce hydrogen through steam catalytic reforming over a sequence of composite catalyst particles Ni/CeO_2_–ZrO_2_ examined in a lab-scale packed bed reactor on hydrogen production performance. Effect of the ratio of water/bio-oil, the reaction experimentally, and numerically were studied [74].

Dehydrogenation of ethane took place in millisecond catalytic wall reactors. The heat transfer between the endothermic and exothermic reaction streams occurs at a high rate once the chemical reaction occurs on the thin wall on opposite sides. The performance of the reactor was stable and not affected by time [75]. Phenol catalytic steam reformer over bimetallic nickel–cobalt catalysts on various supports efficiently produces hydrogen. The catalyst shows conversion of phenol reached about 96%, and hydrogen yield was 80% at 650 °C. The catalyst particle (composed of 5 wt% nickel and 5 wt% cobalt on the \support) was tested in a fixed microreactor bed at a temperature of 500 to 800 °C [76]. High heat transfer occurs between endothermic and exothermic reactions inside catalytic wall reactors.

The reaction taking place on the thin wall on opposite sides removes the resistance to heat transfer; accordingly, they are more efficient and compact than other reactors. The reactor conversion achieves 95% of methane to produce synthesis gas. The residence time is about 70 milliseconds and the steam-to-methane ratio [77]. A review of the tar catalytic steam reforming enhanced the production of hydrogen from the gasification of biomass. The study considered development in tar production and analysis-supported metal catalysts [78]. The methane steam reforming at a low temperature supported nickel-based catalysts for hydrogen production where the coke formation was low [79]. Catalytic combustion of methane was carried out in a compact multichannel reformer. The heat was provided by methane oxidation in the combustor catalytic reaction on both sides of the reformer. The chemical reaction is highly endothermic, and the method is cost-effective. The hydrogen production rate with methane catalytic oxidation is sufficient to produce a 30 W fuel cell [80].

The CFD modeling was developed to simulate the methane industrial-scale steam reformer. The model was based on predicted chemical reactions and transport phenomena. The ANSYS Fluent software package was used to solve the established mathematical model. The CFD model predictions produced by simulating an industrial-scale reformer were in good agreement with typical plant data reported in the literature for the same steam methane reformer system [81]. A CFD model was developed to describe the photocatalytic water splitting in a multiphase flow reactor with a solar concentrator employed for photocatalytic water breaking to produce hydrogen. The CFD combined the radiation field, the multiphase flow, and the reaction kinetics. The integrated model was helpful for the improvement of the design of the photocatalytic reactor and the system’s optimum operation conditions [82]. Computational fluid dynamics (CFD) modeling of methane steam reforming reactor (150 mm length and 100 mm radius) occupied with nickel-based catalyst, was developed to study the influence of modeling geometric dimensionality (1D, 2D, and 3D computational domains) on the system performance. The results disclosed the reformer product composition, conversion, temperature profile, and diffusion flux. The ANSYS Fluent commercial software package performed the comparative analysis. The simulation results did not show any significant differences between 3D and 2D geometry, neither 3D nor 1D for high residence time. Accordingly, the results concluded that for engineering modeling and simulation purposes, and a ratio of more than 8 kg catalyst per mol CH_4_, 1D modeling is sufficient. The 2D and 3D modeling can make noticeable differences in modeling predictions for small residence time values and relative length [83].

### 3.7. Membrane-Assisted Packed Bed Reactor

A CFD model was performed to investigate the performance of steam ethanol reforming. The developed mathematical model was employed to explore the reformer system on bed volume range (macroscale) and particle scale (microscale) inside a membrane fixed bed reactor (Figure 7). The model evaluated the influence of bed porosity, particle size on diffusion, and performance of the reforming process. Results disclosed that for large catalyst particle size, the membrane separation limits the external distribution of the gas species in the bed [84]. At low reactor heights, internal gas diffusion significantly impacts the reaction rate [85].

Control and CFD modeling of methane steam reforming of an industrial scale reactor, with feedback control schemes, was performed to control the hydrogen mole fraction in the product stream. ANSYS Fluent was employed to simulate the industrial reformer with realistic geometry to pretend the chemical reaction and transport phenomena. The CFD simulation predictions disclose that the three different feedback control schemes (P, PI, and PID) can drive the hydrogen outlet mole fraction to the setpoint value. Compared to open-loop control, the feed disturbance of a tube side improves the process dynamics [86]. The heat losses and flow distribution influence on the methanol steam reforming were done using a three-dimensional CFD simulation in a microchannel and micro slits reactor. Results revealed high percentages of heat loss of the energy provided to the microreactor [87]. Derivation of intrinsic steam methane reforming kinetics was performed on a nickel–ceria fuel cell anode. Experimentally, the partial pressures of steam, hydrogen, and methane on the steam methane reforming were investigated. Experimental data in a typical plug flow reactor model parameterized different kinetic rate equations [88]. Heterogeneous, two multiscale, and non-isothermal dimensional models were developed for packed bed reactors of industrial scale where methane steam reforming took place. The model accounts for radial and axial dispersion in the tubes of the reactor. The model also accounts for heat and mass transfer resistances at the solid–fluid interphase boundary [89].

### 3.8. Double Coupled Reactor

A CFD model studied the novel thermally double-coupled reactor (TDCR). The heat evolved from the exothermic synthesis of methanol in the inner tube (CO_2_ + H_2_) to provide the endothermic glycerol reforming reaction in the annulus (glycerol + steam). The glycerol aqueous phase reaction was a source of hydrogen production (Figure 8). The thermally double-coupled reactor was analyzed using the CFD model. The catalyst was Pt/Al_2_O_3_ at 200 to 250 °C and a pressure of 50 to 80 bars. The standard co-current arrangement mode provided a uniform temperature profile compared to the countercurrent mode. Cold and hot spots were observed in the countercurrent mode configuration. Changing operating conditions affected reactor performance [90].

### 3.9. Catalytic Plate Reactor

A 2D mathematical model was developed to describe the steam methane reforming and combustion of methane over a portion layer coated with the catalyst in a plate reactor (Figure 9). The numerical model describes steam methane reforming and combustion over coated catalyst layers in a plate reactor to produce hydrogen. The CFD model described the reactor performance. The length of the reactor, the steam-to-carbon ratio, and the catalyst bed temperature were used to evaluate the hydrogen production rate, methanol conversion, and carbon monoxide concentration in the produced mixture. The segmented coated catalysts consumed 66% less combustion catalyst than the conventional catalytic plate reactor while converting methane and hydrogen yield in methane steam reforming. The maximum temperature of the plate reactor and the thermal hot spots dropped significantly in the modified reactor with segmented coated catalysts.

### 3.10. Traditional versus Membrane Reactor

**Figure 10:**
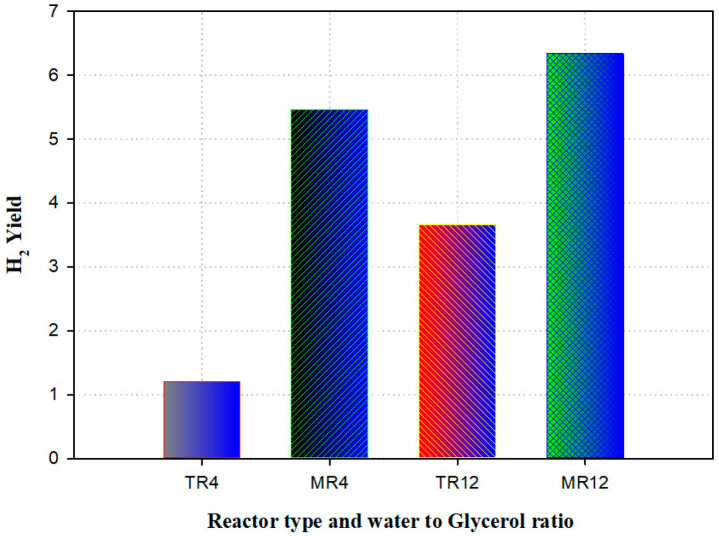
Hydrogen yield generated from traditional (TR) versus membrane reactor (MR) at various temperatures and water-to-glycerol feed ratio (4 and 12). Reproduced from [92].

A model was established to predict the presence of a membrane reactor against a traditional reactor (TR) for the manufacturing of hydrogen-utilizing steam reforming of glycerol. The experimental data validated the mathematical model. The outcomes were in good agreement, which was validated against the practical result. Compared to the traditional reactor, simulation results revealed that the membrane (with the thickness of 15 mm) reactor displayed an improvement in the H2 yield of 74%. The maximum amount of H_2_ produced was examined for the membrane reactor and traditional reactor (Figure 10). The results disclosed that a rise of 15% was detected in the membrane reactor [92].

## 4. Conclusions

Various CFD models were established to describe hydrogen production in different types of catalytic steam reforming reactors. Specific numerical models considered catalyst type and catalyst deactivation. The CFD model was used to examine the sedimentation of coke inside a packed bed reactor and onto a single catalyst particle. Coke deposition can easily deactivate catalyst particles with more significant porosity and smaller particle diameter, which is more effective near the walls of the fixed bed. By contrast, other studies disclosed that catalysts with small particle sizes and extensive porosity help increase hydrogen diffusion and enhance the membrane reactor performance. Accordingly, future work is likely to consider more experimental work to validate the diffusion effect considering catalyst deactivation with coke deposition. The type of catalyst is crucial; compared to a commercial nickel-based catalyst, experimental results revealed the Ni/CeO_2_–ZrO_2_ catalyst had superior catalytic activity for hydrogen production by steam reforming bio-oil. Ansys Fluent and Comsol Multiphysics were the most popular commercial software packages used for CFD simulations of the hydrogen production steam reforming processes.

## Figures and Tables

**Table 1:** List of the main CFD simulation of the steam reforming for hydrogen production.

Type of System	Process	Software	Ref.
Baffled membrane reactor	Hydrogen production by steam methane reforming	Ansys Fluent	[12]
Impact of catalyst bed density	Glycol steam reforming for hydrogen production	Blender 3D	[13]
Compact steam methane reforming process standalone application	Compact steam methane reforming process		[14]
Microchannel reformer	Steam methane reforming	Ansys Fluent	[15]
Catalytic membrane reactor	Methanol steam reforming	COMSOL Multiphysics^®^ 5.6	[16]
Autothermal reactor	Steam methane reforming and methane combustion	SIMPLE algorithm with Fortran programming language	[17]
Steam methane reactor installed in a pilot plant	Steam methane reforming	ANSYS Fluent 19.0	[18]
Fuel cell application	Hydrogen production from PEM fuel cell		[19]
membrane-integrated reformer reactor (MRR)	Methane reforming and hydrogen separation	ANSYS Fluent	[20]
Radiation section of the top industrial steam methane reforming reactor	Steam methane reforming	Single program multiple data passing model	[21]
Wire mesh honeycomb catalytic module	Diesel reforming for hydrogen production	COMSOL Multiphysics 5.5	[22]
Hydrogen generation for an on-site hydrogen refueling station (HRS)	Steam methane reforming	Ansys Fluent	[23]
Membrane reactor	Methane steam reforming	User-defined function (UDF)	[24]
Heat flow inside a catalyst particle of catalytic reactor	Steam methane reforming	Ansys Fluent	[25]
Hydrogen permeable membrane reactor	CO_2_ reforming of CH_4_	COMSOL Multiphysics modeling software v5.2	[26]
Catalytic wall reactors (Rh–Pd/CeO_2_ catalyst)	Ethanol steam reforming	COMSOL Multiphysics 5.4	[27]
Catalytic reactor (Cu–Zn/γ-Al_2_O_3_ catalyst)	Dimethyl ether steam reforming	COMSOL 5.2 software	[28]
Fixed bed reactor (effect of radiation)	Steam methane reforming	STAR-CCM+ from Siemens PLM	[29]
Preheated Ni-based catalyst bed reactor	Steam methane reforming	Ansys Fluent	[30]
Pd–Ag membrane rector	Glycerol steam reforming process	COMSOL software	[31]
Catalytic bed reactor and ethanol burner	Ethanol steam reforming system	COMSOL Multiphysics	[32]
Fluidized bed reactor	Sorption-enhanced crude glycerol steam reforming process	Platform of MFIX CFD code	[33]
Catalytic membrane reactor	Ethanol steam reforming process	Comsol Multiphysics	[34]
Sorption-enhanced palladium membrane reactor	Methane steam reforming	Ansys commercial CFD code CFX	[35]
Wall catalytic steam reforming	Steam reforming of aviation kerosene	Fluent	[36]

## Data Availability

All data generated or analyzed during this study are included in this published article. Additional explanations or data are available from the corresponding author upon reasonable request.
